# Long-term safety of cyclical rozanolixizumab in patients with generalized myasthenia gravis: Results from the Phase 3 MycarinG study and an open-label extension

**DOI:** 10.1177/22143602241308181

**Published:** 2025-03-04

**Authors:** Ali A Habib, Artur Drużdż, Julian Grosskreutz, Renato Mantegazza, Sabrina Sacconi, Kimiaki Utsugisawa, Tuan Vu, John Vissing, Maryam Gayfieva, Irene Pulido-Valdeolivas, Thaïs Tarancón, Franz Woltering, Vera Bril, on behalf of the MycarinG and MG0007 study investigators

**Affiliations:** 1 MDA ALS and Neuromuscular Center, Department of Neurology, University of California, Irvine, Orange, CA, USA; 2 Department of Neurology, Municipal Hospital Poznań, Poznań, Poland; 3 Precision Neurology of Neuromuscular Diseases, Department of Neurology, University of Lübeck, Lübeck, Germany; 4 Fondazione Istituto di Ricovero e Cura a Carattere Scientifico, Istituto Nazionale Neurologico Carlo Besta, Milan, Italy; 5 Université Côte d’Azur, Peripheral Nervous System and Muscle Department, Pasteur 2 Hospital, Centre Hospitalier Universitaire de Nice, Nice, France; 6 Department of Neurology, Hanamaki General Hospital, Hanamaki, Japan; 7 Department of Neurology, University of South Florida, Morsani College of Medicine, Tampa, FL, USA; 8 Copenhagen Neuromuscular Center, Department of Neurology, Rigshospitalet, University of Copenhagen, Copenhagen, Denmark; 9 UCB, Slough, UK; 10 UCB, Madrid, Spain; 11 UCB, Monheim, Germany; 12 University Health Network, Toronto, Canada

**Keywords:** neonatal Fc receptor, generalized myasthenia gravis, monoclonal antibody, rozanolixizumab, safety, MuSK, myasthenia gravis, ACh receptor

## Abstract

**Background:**

Generalized myasthenia gravis (gMG) is a rare, chronic, fluctuating and heterogeneous autoimmune disease requiring lifelong treatment. The Phase 3 MycarinG study demonstrated the efficacy and safety of one 6-week cycle of weekly rozanolixizumab in adult patients with gMG. Open-label extension studies demonstrated consistent symptom improvement over additional treatment cycles.

**Objective:**

To present findings from pooled analyses on the long-term safety of repeated cycles of rozanolixizumab.

**Methods:**

Data from the Phase 3 randomized MycarinG study (NCT03971422) and the ongoing open-label extension study MG0007 (NCT04650854) were pooled to assess safety outcomes during cyclical treatment, including incidence of any treatment-emergent adverse events (TEAEs), severe TEAEs, serious TEAEs and TEAEs leading to discontinuations. Additional analyses were performed for TEAEs, including headache, infections, and hypersensitivity reactions.

**Results:**

At data cutoff (July 8, 2022), a total of 188 patients in MycarinG and MG0007 had received ≥1 treatment cycle with rozanolixizumab; total time in studies was 174.71 patient-years. Overall, 169/188 (89.9%) patients experienced any TEAE: 89/188 (47.3%) experienced any headache (including migraine, migraine with aura); 85/188 (45.2%) experienced an infection; 25/188 (13.3%) experienced a hypersensitivity reaction. One patient experienced an event of aseptic meningitis. The majority of AEs were mild-to-moderate in intensity, and incidence did not increase with repeated cyclic treatment. A total of 50/188 (26.6%) patients experienced severe TEAEs, the most common of which were MG worsening in 4/133 (3.0%) and 7/131 (5.3%) patients in the rozanolixizumab 7 mg/kg and rozanolixizumab 10 mg/kg groups, respectively, MG crisis in 0 and 4/131 (3.1%) patients, and headache in 1/133 (0.8%) and 7/131 (5.3%) patients.

**Conclusions:**

These pooled results, representing 174.71 patient-years in the studies, demonstrate that treatment with rozanolixizumab in patients with gMG was well tolerated, and TEAEs were consistent and did not increase in incidence over repeated cycles in this patient population.

## Plain language summary

Generalized myasthenia gravis (gMG) is an autoimmune disease which may require lifelong treatment. A Phase 3 study called MycarinG has shown that one 6-week cycle of rozanolixizumab in adult patients with gMG had few side effects and reduced the severity of the disease. To further explore the safety of rozanolixizumab over additional treatment cycles, data from MycarinG and a long-term study, MG0007, were combined.

MycarinG was a randomized, double-blind, placebo-controlled study in which patients with acetylcholine receptor or muscle-specific tyrosine kinase receptor autoantibody-positive gMG received subcutaneous infusions of rozanolixizumab 7 mg/kg, rozanolixizumab 10 mg/kg or placebo once weekly for 6 weeks, followed by an 8-week observation period. MG0007 is an ongoing, open-label extension study in which patients receive repeated 6-week cycles of rozanolixizumab given when a patient's symptoms worsened after they stopped treatment. Data from both studies were combined to assess safety outcomes.

As of July 8, 2022, 188 patients in MycarinG and MG0007 had received ≥1 treatment cycle with rozanolixizumab. Overall, 169 (89.9%) patients experienced a side effect. Headaches were experienced by 89 (47.3%) patients; 85 (45.2%) patients experienced an infection; and 25 (13.3%) patients experienced a hypersensitivity reaction. Most side effects were mild-to-moderate in intensity, and they did not become more frequent with repeated cycles of rozanolixizumab. Severe side effects were experienced by 50 (26.6%) patients. Severe events related to MG worsening or MG crisis were experienced by 4 (3.0%) patients receiving rozanolixizumab 7 mg/kg and 11 (8.4%) patients receiving rozanolixizumab 10 mg/kg, and severe headaches were experienced by 1 (0.8%) patient receiving rozanolixizumab 7 mg/kg and 7 (5.3%) patients receiving rozanolixizumab 10 mg/kg.

These results showed that treatment with rozanolixizumab in patients with gMG was well tolerated and that the incidence of side effects did not increase with repeated cycles of treatment

## Introduction

Generalized myasthenia gravis (gMG) is a rare, fluctuating and heterogeneous autoimmune disease in which pathogenic autoantibodies trigger impairments in neuromuscular transmission leading to fatigue and muscle weakness that can be life threatening.^1^ Conventional therapy for gMG is aimed at symptomatic relief and is individualized and guided by factors that include disease severity, response to previous treatments, antibody status and comorbidities.^2^ Broad immunotherapies such as corticosteroids and non-steroidal immunosuppressive treatments are a cornerstone of gMG treatment; however, these agents are often associated with adverse events (AEs), an increased susceptibility to infections and, with immunosuppressants, a delayed onset of action.^2^ Acute disease exacerbations and refractory gMG are treated with plasma exchange (PLEX) or intravenous immunoglobulin (IVIg); these therapies are associated with a significant burden of care on patients due to AEs and lengthy administration times under physician supervision.^1–3^

Rozanolixizumab is a humanized immunoglobulin (Ig) G4 monoclonal antibody that targets the neonatal Fc receptor (FcRn).^4^ FcRn prolongs the half-life of plasma IgG by preventing IgG degradation in lysosomes. Blockade of FcRn is recognized as a treatment target for IgG autoantibody-mediated autoimmune disorders, as it facilitates accelerated IgG degradation by the lysosomal pathway, including pathogenic IgG.^4–6^ FcRn also controls serum albumin homeostasis^7^; rozanolixizumab has been specifically designed to block the IgG (Fc) binding site but not the albumin binding site.^4^

Results from the Phase 3 MycarinG study (NCT03971422) demonstrated that a single 6-week cycle of weekly rozanolixizumab treatment in patients with acetylcholine receptor (AChR) autoantibody-positive (Ab+) or muscle-specific tyrosine kinase (MuSK) Ab+ gMG improved myasthenia gravis (MG)-specific outcomes and was generally well tolerated and had an acceptable safety profile.^6^ A subsequent analysis of pooled data from MycarinG and its open-label extension studies (MG0004/NCT04124965; MG0007/NCT04650854) found the clinical improvements to be consistent across repeated symptom-driven cycles of rozanolixizumab treatment.^8^ Here, we provide further data on the long-term safety of cyclic rozanolixizumab treatment in MycarinG and MG0007, including additional analyses on headache, infections, hypersensitivity and injection-site reactions.

## Materials and methods

### Study design

Full study designs for MycarinG and MG0007 are reported elsewhere.^6,8^ In brief, MycarinG was a randomized, double-blind, placebo-controlled study in patients with AChR Ab+ or MuSK Ab+ gMG, in which patients received subcutaneous infusions of rozanolixizumab 7 mg/kg, rozanolixizumab 10 mg/kg or placebo once weekly for 6 weeks, followed by an 8-week observation period.^6^ Patients who completed the observation period of MycarinG, or required (but chose not to receive) rescue therapy due to disease worsening during the observation period of MycarinG, could roll over into the open-label extension studies to receive rozanolixizumab as chronic weekly treatment in MG0004 or cyclic treatment in MG0007.^6^ Data from MG0004 will be described in a separate publication.

MG0007 is an ongoing, randomized, open-label extension study in which patients receive repeated 6-week cycles of rozanolixizumab.^8^ Once MG0007 was initiated at the clinical site, eligible patients from MycarinG moved directly into MG0007. After an initial treatment cycle, patients received symptom-driven cycles based on symptom worsening. A symptom-driven treatment cycle is defined as a treatment cycle received by a patient following symptom worsening based on the investigator's discretion, with ≥2.0-point increase in Myasthenia Gravis Activities of Daily Living (MG-ADL) score or ≥3.0-point increase in quantitative myasthenia gravis (QMG) score used as examples in the protocol. The treatment period (time from first infusion to 1 week after the final infusion in a cycle) was followed by a 16-week observation period, with scheduled visits, and a further non-treatment period of variable duration during which patients were assessed for symptom worsening.

### Study population: Cyclic rozanolixizumab treatment

The safety of repeated 6-week cycles of rozanolixizumab was assessed in all patients from MycarinG and MG0007 (up to an interim data cutoff of July 8, 2022) who received ≥1 dose of rozanolixizumab in any 6-week treatment period and had a follow-up period of up to 8 weeks (safety pool, defined as all rozanolixizumab-treated study participants who had undergone at least 1 treatment cycle and an [up to] 8-week follow-up period starting from the last infusion).

Immunogenicity was analyzed in patients who received only cyclic rozanolixizumab treatment, with no chronic weekly treatment interruption (immunogenicity pool; i.e., excluding patients who entered MG0004 before MG0007).

Patients were analyzed by the most recent dose received in the pooled analysis; those who received both 7 mg/kg and 10 mg/kg in any cycle were included in analysis of both treatment groups when treatment groups were analyzed individually. Treatment-emergent adverse events (TEAEs) were attributed to the most recently received dose level at the time of AE onset.

Pre-specified additional analyses were performed for severe headache, severe diarrhea, severe vomiting, severe abdominal pain and opportunistic infection.

### Assessments

Safety outcomes reported in this paper are TEAEs, serious AEs (defined as resulted in death, was life-threatening, required inpatient hospitalization or prolongation of existing hospitalization, resulted in persistent disability/incapacity, was a congenital anomaly/birth defect or was considered an important medical event) or TEAEs leading to discontinuation. Severe AEs were defined as Grade 3–5 AEs using Common Terminology Criteria for Adverse Events (CTCAE) version 5 for severity. Additional analyses were conducted for headache, infections including opportunistic infections, hypersensitivity reactions including anaphylactic reactions, injection-site reactions, AEs related to gastrointestinal (GI), hepatic and renal disorders and AEs related to lipids, albumin and total protein metabolism.

The immunogenic potential of rozanolixizumab and risk of immunogenicity-related clinical consequences, in particular safety, were assessed via monitoring of anti-drug antibody (ADA) formation.

### Statistical analysis

Data were analyzed (with SAS version 9.4 or later) using frequency analyses of dichotomous and categorical variables displaying the number of observations and percentages; for continuous variables, the number of observations, mean, standard deviation (SD), median, minimum and maximum values are provided. AEs were classified using the Medical Dictionary for Regulatory Activities (MedDRA) version 24.0.

## Results

### Patient baseline characteristics and treatment exposure

A total of 188 patients in MycarinG and MG0007 received ≥1 treatment cycle (at least 1 cycle of rozanolixizumab 7 mg/kg, n = 128; at least 1 cycle of rozanolixizumab 10 mg/kg, n = 131), with a safety follow-up period of up to 8 weeks. Disease characteristics at baseline were generally balanced across treatment groups (Table 1). There was a lower proportion of patients with Myasthenia Gravis Foundation of America Class IIIa MG in the rozanolixizumab 7 mg/kg group compared with the rozanolixizumab 10 mg/kg group (29.8% and 43.6%, respectively) and a higher proportion of patients with prior thymectomy in the rozanolixizumab 7 mg/kg group compared with the rozanolixizumab 10 mg/kg group (45.7% versus 34.0%). Across both treatment groups, there was a higher proportion of AChR Ab+ patients (approximately 90%) than MuSK Ab+ patients (9/94 [9.6%] in each treatment group). At MycarinG baseline, the majority of patients (96.3%) received at least 1 medication to treat gMG (most commonly cholinesterase inhibitors; systemic corticosteroids and/or immunosuppressants); use of baseline medication to treat gMG was balanced across both treatment groups (Table 1).

**Table 1.** Baseline characteristics.

	RLZ 7 mg/kg* (N = 94)	RLZ 10 mg/kg* (N = 94)	RLZ total (N = 188)
Age, years, mean (SD)	53.1 (14.9)	52.0 (17.6)	52.5 (16.3)
Sex, female, n (%)	56 (59.6)	55 (58.5)	111 (59.0)
Body weight, n (%)			
<50 kg	9 (9.6)	2 (2.1)	11 (5.9)
50 to <70 kg	22 (23.4)	37 (39.4)	59 (31.4)
70 to <100 kg	44 (46.8)	32 (34.0)	76 (40.4)
≥100 kg	19 (20.2)	23 (24.5)	42 (22.3)
BMI (kg/m^2^), mean (SD)	27.5 (6.5)	28.1 (6.4)	27.8 (6.5)
Geographic region, n (%)			
North America	30 (31.9)	24 (25.5)	54 (28.7)
Europe	52 (55.3)	62 (66.0)	114 (60.6)
Asia (excl. Japan)	4 (4.3)	3 (3.2)	7 (3.7)
Japan	8 (8.5)	5 (5.3)	13 (6.9)
Race, n (%)			
Asian	12 (12.8)	9 (9.6)	21 (11.2)
Black	0	4 (4.3)	4 (2.1)
Native Hawaiian or other Pacific Islander	0	1 (1.1)	1 (0.5)
White	58 (61.7)	69 (73.4)	127 (67.6)
Missing^#^	24 (25.5)	11 (11.7)	35 (18.6)
Mean age at initial MG diagnosis, years (SD)	45.5 (16.2)	43.2 (19.8)	44.3 (18.0)
Median duration of disease, years (range)	5.8 (0.4–48.9)	5.7 (0.1–46.4)	5.7 (0.1–48.9)
MGFA disease class at baseline, n (%)			
Class IIa	20 (21.3)	16 (17.0)	36 (19.1)
Class IIb	25 (26.6)	14 (14.9)	39 (20.7)
Class IIIa	28 (29.8)	41 (43.6)	69 (36.7)
Class IIIb	17 (18.1)	21 (22.3)	38 (20.2)
Class IVa	4 (4.3)	2 (2.1)	6 (3.2)
Myasthenia crisis, n (%)			
Yes	25 (26.6)	28 (29.8)	53 (28.2)
No	68 (72.3)	65 (69.1)	133 (70.7)
Missing	1 (1.1)	1 (1.1)	2 (1.1)
Baseline gMG medication, n (%)			
Corticosteroids for systemic use	56 (59.6)	64 (68.1)	120 (63.8)
Immunosuppressants	45 (47.9)	52 (55.3)	97 (51.6)
Cholinesterase inhibitors	80 (85.1)	82 (87.2)	162 (86.2)
Thymectomy at baseline, n (%)	43 (45.7)	32 (34.0)	75 (39.9)
Mean MG-ADL score at baseline (SD)	8.3 (3.7)	8.4 (2.9)	8.3 (3.4)
Mean QMG score at baseline (SD)	15.4 (3.6)	15.8 (3.6)	15.6 (3.6)
Mean total IgG at baseline, g/L (SD)	10.2 (2.9)	9.8 (2.7)	10.0 (2.8)
MuSK autoantibody-positive, n (%)	9 (9.6)	9 (9.6)	18 (9.6)
AChR autoantibody-positive, n (%)	84 (89.4)	86 (91.5)	170 (90.4)

Safety pool. *The allocation of patients to treatment group was based on the maximum dose received during the first rozanolixizumab treatment cycle, although patients may have switched rozanolixizumab doses within or between subsequent cycles. ^#^Data on race were not permitted to be collected in certain countries.

AChR: acetylcholine receptor; BMI: body mass index; gMG: generalized myasthenia gravis; IgG: immunoglobulin G; MG-ADL: Myasthenia Gravis Activities of Daily Living; MGFA: Myasthenia Gravis Foundation of America; MuSK: muscle-specific kinase; QMG: Quantitative Myasthenia Gravis; RLZ: rozanolixizumab; SD: standard deviation; TEAE: treatment-emergent adverse event.

Up to data cutoff (July 8, 2022), 678 cycles of rozanolixizumab had been initiated in these 188 patients (mean [SD] number of complete and incomplete initiated cycles per patient 3.6 [2.2], balanced across both treatment groups). Total time in studies was 174.71 patient-years, with a mean (SD) time in the studies of approximately 1 year (339.2 [150.2] days; median 368.0 [range 44–599] days). A total of 24/188 (12.8%) patients had started at least 7 cycles and 3/188 (1.6%) patients had started 9 cycles. Ninety-seven of the 188 patients (51.6%), with a combined 121.1 patient-years of exposure, had undergone more than 1 year of study participation (median cycles initiated within the first year: 4 [range: 1–7]). Among these 97 patients, 51 (52.6%) initiated ≥5 cycles and 37 (38.1%) initiated ≥6 cycles of rozanolixizumab treatment across the total duration of their participation in the study.

### TEAEs in patients receiving cyclic rozanolixizumab treatment

Overall, 169/188 (89.9%) patients in the cyclic treatment safety pool experienced any TEAE (Table 2), with a higher incidence reported in the rozanolixizumab 10 mg/kg group than the rozanolixizumab 7 mg/kg group, which did not increase with repeated treatment cycles. The most common TEAEs overall were headache, diarrhea and pyrexia (Table 2).

**Table 2.** Overview of adverse events.

	All cycles (N = 188)*	Cycle 1 (N = 188)*	Cycle 2 (N = 143)*	Cycle 3 (N = 113)*	Cycle 4 (N = 92)*	Cycle 5 (N = 63)*	Cycle 6 (N = 43)*
Any TEAEs, n (%)^#^	169 (89.9)	147 (78.2)	100 (69.9)	67 (59.3)	53 (57.6)	46 (73.0)	28 (65.1)
Headache	87 (46.3)	69 (36.7)	34 (23.8)	21 (18.6)	16 (17.4)	10 (15.9)	9 (20.9)
Diarrhea	54 (28.7)	36 (19.1)	11 (7.7)	7 (6.2)	9 (9.8)	7 (11.1)	3 (7.0)
Pyrexia	34 (18.1)	25 (13.3)	9 (6.3)	2 (1.8)	5 (5.4)	2 (3.2)	3 (7.0)
Nausea	28 (14.9)	15 (8.0)	9 (6.3)	6 (5.3)	4 (4.3)	4 (6.3)	-
COVID-19	26 (13.8)	4 (2.1)	10 (7.0)	5 (4.4)	4 (4.3)	1 (1.6)	2 (4.7)
Arthralgia	21 (11.2)	10 (5.3)	4 (2.8)	2 (1.8)	6 (6.5)	-	2 (4.7)
Blood IgG decreased	20 (10.6)	4 (2.1)	7 (4.9)	5 (4.4)	7 (7.6)	5 (7.9)	3 (7.0)
MG	18 (9.6)	9 (4.8)	2 (1.4)	2 (1.8)	2 (2.2)	4 (6.3)	-
Vomiting	13 (6.9)	6 (3.2)	2 (1.4)	2 (1.8)	1 (1.1)	1 (1.6)	1 (2.3)
Upper respiratory tract infection	13 (6.9)	3 (1.6)	4 (2.8)	1 (0.9)	1 (1.1)	4 (6.3)	2 (4.7)
Serious TEAEs, n (%)	42 (22.3)	20 (10.6)	9 (6.3)	5 (4.4)	5 (5.4)	6 (9.5)	-
Permanent discontinuation from study due to TEAEs, n (%)	29 (15.4)	13 (6.9)	8 (5.6)	3 (2.7)	4 (4.3)	1 (1.6)	-
Permanent discontinuation of study drug due to TEAEs, n (%)	27 (14.4)	12 (6.4)	8 (5.6)	2 (1.8)	4 (4.3)	1 (1.6)	-
Treatment-related TEAEs, n (%)	111 (59.0)	94 (50.0)	51 (35.7)	26 (23.0)	28 (30.4)	22 (34.9)	18 (41.9)
Severe TEAEs, n (%)	50 (26.6)	23 (12.2)	9 (6.3)	6 (5.3)	8 (8.7)	8 (12.7)	2 (4.7)
Deaths, n (%)	3 (1.6)	-	2 (1.4)	1 (0.9)	-	-	

Safety pool.

*Summaries by cycle are displayed if the total number of patients undergoing the cycle for the for 7 mg/kg and 10 mg/kg rozanolixizumab dose groups combined was ≥10.

^#^ Individual AEs listed are those occurring in ≥5% of patients in the rozanolixizumab combined dose group in any treatment cycle.

AE: adverse event; COVID-19: coronavirus disease 2019; IgG: immunoglobulin G; GI: gastrointestinal; MG: myasthenia gravis; RLZ: rozanolixizumab; TEAE: treatment-emergent adverse event.

There were no apparent differences in TEAE profile across most demographic and disease characteristics, including autoantibody status (AChR and MuSK Ab+ patients). However, the incidence of treatment-related TEAEs was higher in females (70/111 [63.1%]) compared with males (41/77 [53.2%]) and in younger patients (18 to <65 years; 87/140 [62.1%]) compared with elderly patients (≥65 years; 24/48 [50.0%]). TEAE of headaches, in particular, occurred more frequently in female compared with male patients (59/111 [53.2%] and 28/77 [36.4%], respectively) and in younger compared with elderly patients (70/140 [50.0%] and 17/48 [35.4%], respectively).

#### Severe TEAEs

In total, 50/188 (26.6%) patients experienced severe TEAEs (Table 2); the incidence of severe TEAEs was higher in the rozanolixizumab 10 mg/kg group than in the rozanolixizumab 7 mg/kg group. The most frequently reported severe TEAEs were events related to MG worsening/crisis (MG worsening in 4/133 [3.0%] and 7/131 [5.3%] in the rozanolixizumab 7 mg/kg and rozanolixizumab 10 mg/kg groups, respectively and MG crisis in 0 and 4/131 [3.1%] patients) and headache (1/133 [0.8%] and 7/131 [5.3%] patients in the rozanolixizumab 7 mg/kg and rozanolixizumab 10 mg/kg groups, respectively). The incidence of severe TEAEs did not increase with repeated cyclic treatment.

#### Serious TEAEs

Overall, 42/188 (22.3%) patients experienced serious TEAEs (Table 2), of which MG worsening (12/188, [6.4%] patients), MG crisis (4/188, [2.1%]), and COVID-19 (3/188 [1.6%]) occurred in >1 patient. In the individual treatment groups, the incidence of serious TEAEs was higher in the rozanolixizumab 10 mg/kg group (29/131 [22.1% patients]) than in the rozanolixizumab 7 mg/kg group (14/133 [10.5% patients]). Compared with Cycle 1, the incidence of serious TEAEs did not increase with repeated cyclic treatment.

#### TEAEs leading to discontinuation

TEAEs leading to permanent discontinuation from the study occurred in 29/188 (15.4%) patients (Table 2), including 8/133 (6.0%) and 21/131 (16.0%) patients in the rozanolixizumab 7 mg/kg and rozanolixizumab 10 mg/kg groups, respectively. Incidences remained low throughout repeated cycles of treatment (<7%). The most frequently reported TEAEs leading to study discontinuation during repeated cycles across both treatment groups were MG worsening (2/188 and 3/188 patients in the treatment period and follow-up period, respectively) and MG crisis (1 patient each in the treatment and follow-up periods). All patients with TEAEs related to MG worsening who received rescue therapy met protocol-mandated withdrawal criteria.

#### Deaths

Five patients died in MG0007, of whom four had fatal TEAEs and one had a non-TEAE with a fatal outcome after withdrawing from the study; all deaths were considered by investigators as not related to rozanolixizumab treatment. Two patients in the rozanolixizumab 10 mg/kg group died from TEAEs related to COVID-19 (one COVID-19 during Cycle 1, and one COVID-19 pneumonia during Cycle 3); neither patient had been vaccinated against COVID-19. One patient in the rozanolixizumab 7 mg/kg group, aged 69 years and receiving prednisolone, mycophenolate mofetil and amiodarone, was hospitalized with a serious TEAE of pneumonia (no pathogen identified) 57 days after their last rozanolixizumab dose in Cycle 2; the patient subsequently developed acute kidney injury, acute respiratory failure, cardiac failure and acute respiratory distress syndrome and died. One death occurred in the rozanolixizumab 10 mg/kg group after data cutoff and was due to circulatory failure, 51 days after their last infusion. A fatal myocardial infarction occurred in one patient 326 days after their last rozanolixizumab 10 mg/kg dose; the patient had discontinued the study approximately 6 months previously.

#### Headaches

**Figure 1.**
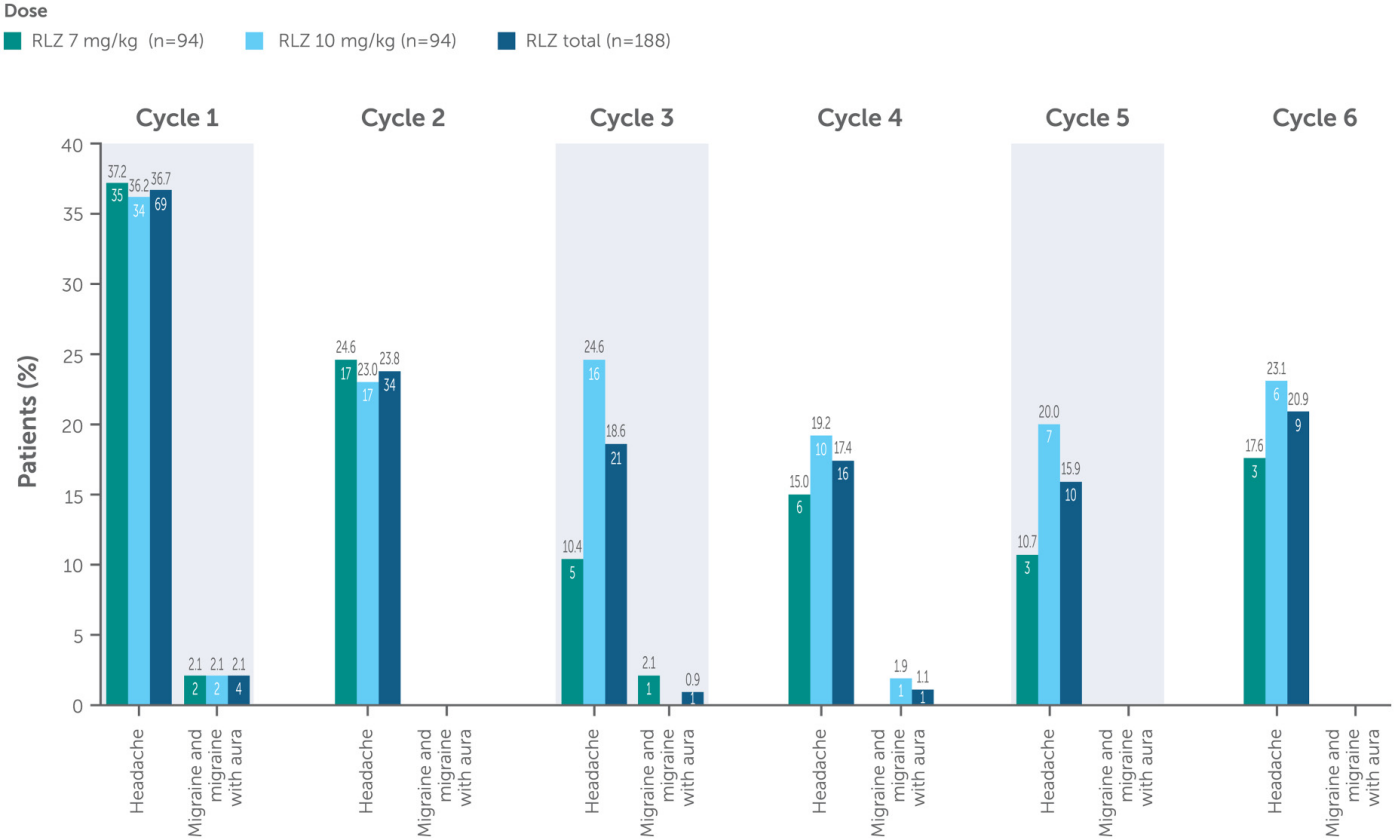
Incidence of headache by treatment cycle (safety pool).

In total, 89/188 patients (47.3%) experienced any headache (including migraine and migraine with aura), which were predominantly mild-to-moderate in intensity with a similar incidence across both dose groups, which was highest in Cycle 1 (Figure 1). Headaches were generally well managed with non-opioid analgesics. Migraine was reported in 5/188 (2.7%) patients and migraine with aura in 1/188 (0.5%) patient. Among these six patients, two had a recorded history of migraine, two had a history of headaches and two had no recorded history of headaches. Eight severe headaches occurred, one (0.8%) in the rozanolixizumab 7 mg/kg group and seven (5.3%) in the rozanolixizumab 10 mg/kg group; all severe headaches occurred during MycarinG (Cycle 1), except for one severe headache in one patient in the rozanolixizumab 10 mg/kg group during MG0007. The severe headaches occurred between 3 to 4 days after the most recent infusion and with a duration ranging between 2 and 4 days. In MycarinG, 5 patients reported severe headache after first infusion, and 2 patients reported severe headache after the second infusion and the third infusion, respectively. In MG0007, one patient experienced severe headache in Cycle 5. In MycarinG, one severe headache in the rozanolixizumab 10 mg/kg group was also classified as serious due to the patient being hospitalized overnight. The event did not lead to treatment interruption, dose change or discontinuation (Table 3). One patient in the rozanolixizumab 7 mg/kg group in MycarinG discontinued the study due to a TEAE of headache.

Data labels show number of patients who experienced the event (inside the bar) and percentage (above the bar).

**Table 3.** Incidence of serious and severe headaches, infections or GI disorders by treatment cycle*.

	All cycles			Cycle 1	Cycle 2	Cycle 3	Cycle 4	Cycle 5	Cycle 6
n (%)	RLZ 7 mg/kg (N = 133)	RLZ 10 mg/kg (N = 131)	RLZ total (N = 188)	RLZ total (N = 188)	RLZ total (N = 143)	RLZ total (N = 113)	RLZ total (N = 92)	RLZ total (N = 63)	RLZ total (N = 43)
Any headache	55 (41.4)	56 (42.7)	89 (47.3)	72 (38.3)	34 (23.8)	21 (18.6)	17 (18.5)	10 (15.9)	9 (20.9)
Serious	0	1 (0.8)	1 (0.5)	1 (0.5)	0	0	0	0	0
Severe	1 (0.8)	7 (5.3)	8 (4.3)	7 (3.7)	0	0	0	0	1 (2.3)
Any infection or infestation	43 (32.3)	54 (41.2)	85 (45.2)	43 (22.9)	24 (16.8)	25 (22.1)	16 (17.4)	19 (30.2)	8 (18.6)
Serious	2 (1.5)	6 (4.6)	8 (4.3)	3 (1.6)	2 (1.4)	1 (0.9)	2 (2.2)	0	0
Severe	1 (0.8)	6 (4.6)	7 (3.7)	2 (1.1)	2 (1.4)	1 (0.9)	2 (2.2)	0	0
Any GI disorder	38 (28.6)	48 (36.6)	73 (38.8)	51 (27.1)	21 (14.7)	14 (12.4)	13 (14.1)	10 (15.9)	5 (11.6)
Serious	2 (1.5)	0	2 (1.1)	2 (1.1)	0	0	0	0	0
Severe	1 (0.8)	2 (1.5)	3 (1.6)	3 (1.6)	0	0	0	0	0

Safety pool.

*Data in the ‘All cycles’ columns are derived by a most recent dose analysis (sum of cycles: 315 for RLZ 7 mg/kg, 363 for RLZ 10 mg/kg and 678 for RLZ total), while data for each individual cycle are derived from a ‘by cycle’ analysis.

GI: gastrointestinal; RLZ: rozanolixizumab.

#### Infections

**Figure 2.**
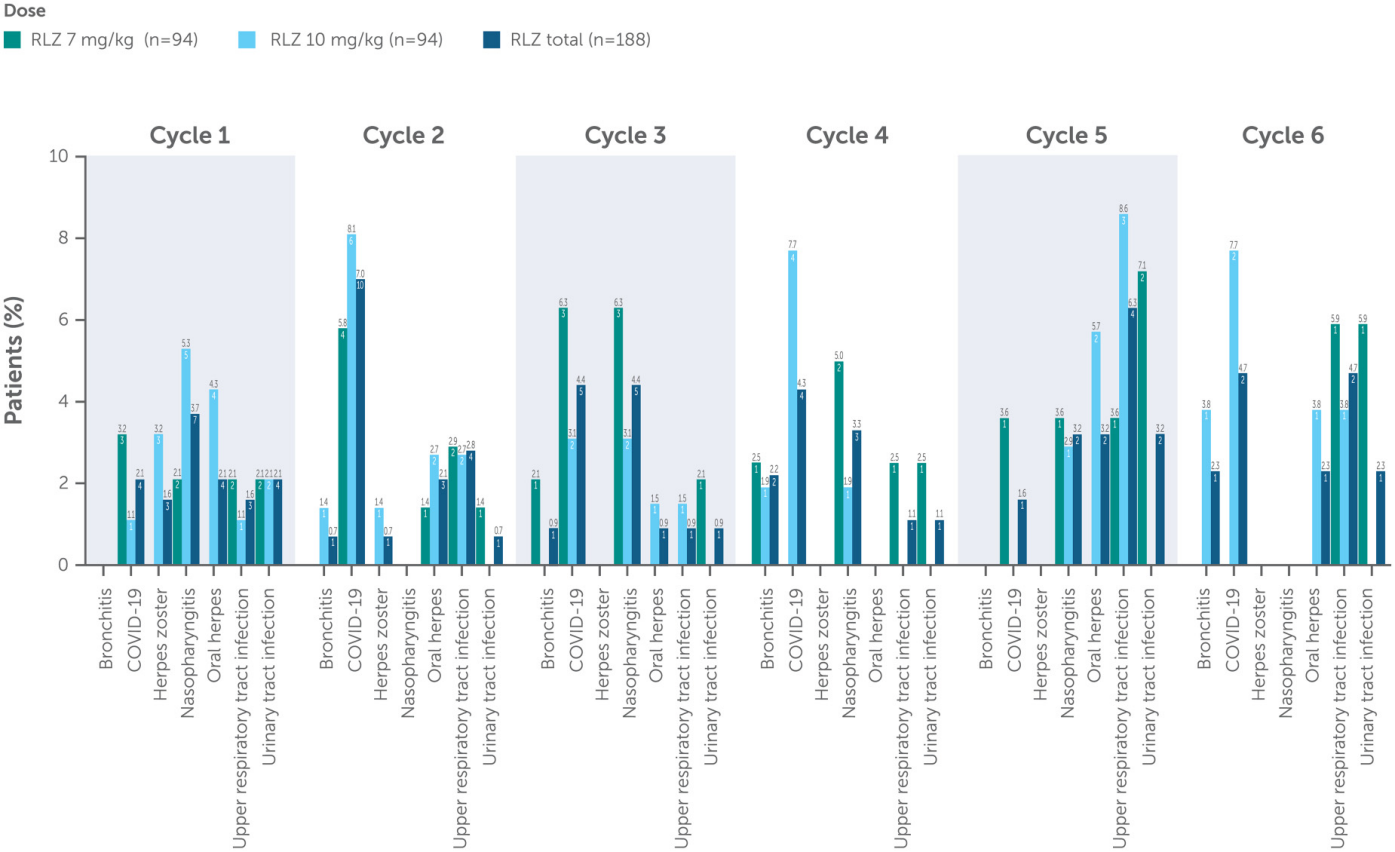
Incidence of infection by treatment cycle.

Over a median 368.0 days in the studies, 85/188 (45.2%) patients reported an infection (Table 3); the incidence of infections did not increase with repeated cycles of treatment. The most common infections in both treatment groups were COVID-19, upper respiratory tract infection, nasopharyngitis and oral herpes (Figure 2). There were no opportunistic infections. The majority of infections were of mild-to-moderate intensity. COVID-19 was the most common infection; however, this was expected as the MycarinG and OLE studies were conducted during the COVID-19 pandemic. Eight (4.3%) patients experienced serious infections (Table 3) – two (1.5%) patients in the rozanolixizumab 7 mg/kg group and six (4.6%) in the rozanolixizumab 10 mg/kg group. Three of these patients died (due to COVID-19, COVID-19 pneumonia, and pneumonia; see earlier section on Deaths) and the remaining five patients recovered fully from their infections. The depth of IgG lowering following rozanolixizumab treatment was not associated with infection risk.

Data labels show number of patients who experienced the event (inside the bar) and percentage (above the bar).

#### Aseptic meningitis

As mentioned above, one patient experienced a serious TEAE of drug-induced aseptic meningitis, which was deemed to be related to rozanolixizumab. The patient received placebo in MycarinG and rolled over into MG0007 where they received rozanolixizumab 10 mg/kg. 1.5 days after the first infusion the patient experienced severe headache with nuchal rigidity, pyrexia, nausea, vomiting and diarrhea, and was hospitalized. Increased white blood cells were detected in the cerebrospinal fluid and no pathogen was identified. Magnetic resonance imaging of the head showed meningeal enhancement in the frontal ventricular system and around the third ventricle. The patient withdrew from the study and fully recovered from severe headache after 5 days.

#### Hypersensitivity and injection-site reactions

Incidence of hypersensitivity-related TEAEs was low (25/188 [13.3%] patients) and did not increase with repeated cyclic treatment. The most common reactions included rash (11/188 [5.9%] patients) and urticaria (3/188 [1.6%] patients); all were mild-to-moderate in intensity except for one case of severe rash. One patient experienced mild swollen tongue during Cycle 1, 1 day (first infusion) and 4 days (fourth infusion) after administration of rozanolixizumab 7 mg/kg. These events were assessed by the investigator as related to study treatment. The patient was treated with cetirizine and both events resolved on the day of onset without treatment discontinuation. This patient also received prophylaxis and did not experience any further events. No serious hypersensitivity or anaphylactic reactions occurred.

In total, 23/188 (12.2%) patients experienced an injection-site reaction, with similar incidence across both dose groups (9.8% of patients in the rozanolixizumab 7 mg/kg group and 9.2% of patients in the rozanolixizumab 10 mg/kg group). The most common injection-site reactions were injection-site erythema (5/188 [2.7%] patients), injection-site bruising (4/188 [2.1%] patients) and injection-site rash (4/188 [2.1%] patients). All injection-site reactions were non-serious and mild-to-moderate in intensity and did not lead to treatment discontinuation.

#### Gastrointestinal disorders

**Figure 3.**
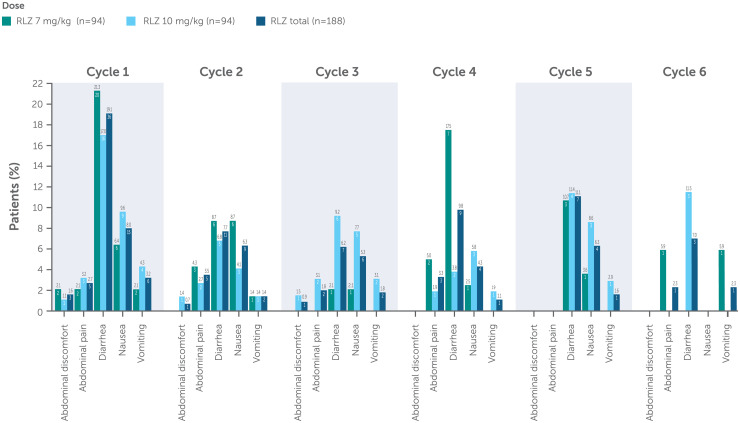
Incidence of GI disorder by treatment cycle.

In total, 73/188 (38.8%) patients experienced a GI disorder (Table 3). Incidence was similar across both dose groups (Figure 3), with the most common events reported as diarrhea and nausea. Serious GI disorders occurred in 2 (1.1%) patients (gastritis and vomiting, 1 patient each), both in the rozanolixizumab 7 mg/kg group (Table 3). Severe GI disorders were reported in 3 (1.6%) patients; 1 patient in the rozanolixizumab 7 mg/kg group experienced severe vomiting, and 2 patients in the rozanolixizumab 10 mg/kg group experienced severe diarrhea. Compared with Cycle 1, the incidence of any GI disorder did not increase with repeated cyclic treatment. There were no reports of severe abdominal pain.

Numbers outside the bars are given as percentage. The numbers inside the bars are the number of patients. GI: gastrointestinal.

#### Other pre-specified treatment-emergent adverse events

Assessment of TEAEs related to hepatic and renal function, abnormalities in lipids and albumin and review of laboratory data, including hepatic and kidney parameters, did not indicate a specific risk of hepatotoxicity or drug-induced liver injury, renal toxicity or effect on lipid profile with rozanolixizumab treatment.

A total of 13 TEAEs of drug-related hepatic disorders were experienced by 9/188 (4.8%) patients. The majority of these TEAEs were mild; none were serious, and one was severe. The one severe event was liver function test increase in a patient in the rozanolixizumab 10 mg/kg group that was judged to be not related to rozanolixizumab treatment. The events reported in >1 patient were increases in alanine aminotransferase, aspartate aminotransferase, blood bilirubin and liver function test each in 2/131 (1.5%) patients in the rozanolixizumab 10 mg/kg group.

Three (1.6%) patients with pre-existing medical conditions and/or low estimated glomerular filtration rate at baseline reported TEAEs of renal impairment, all in the rozanolixizumab 10 mg/kg group: all were mild or moderate, and none were considered related to rozanolixizumab treatment.

TEAEs related to increase in lipid levels were experienced by 8 (4.3%) patients including 3 (2.3%) patients in the rozanolixizumab 7 mg/kg group and 5 (3.8%) in the rozanolixizumab 10 mg/kg group. In MG0007, mean lipid levels remained stable over repeated cycles.

During MycarinG and MG0007, there was a minimal and not clinically meaningful reduction in mean albumin levels within both treatment groups. During MG0007, mean reductions ranged between −2.7 g/L and −1.1 g/L in the 7 mg/kg group and between −3.3 g/L and −0.5 g/L in the 10 mg/kg group. Furthermore, no patient experienced TEAEs related to reductions in albumin or total protein levels.

### Immunogenicity

**Figure 4.**
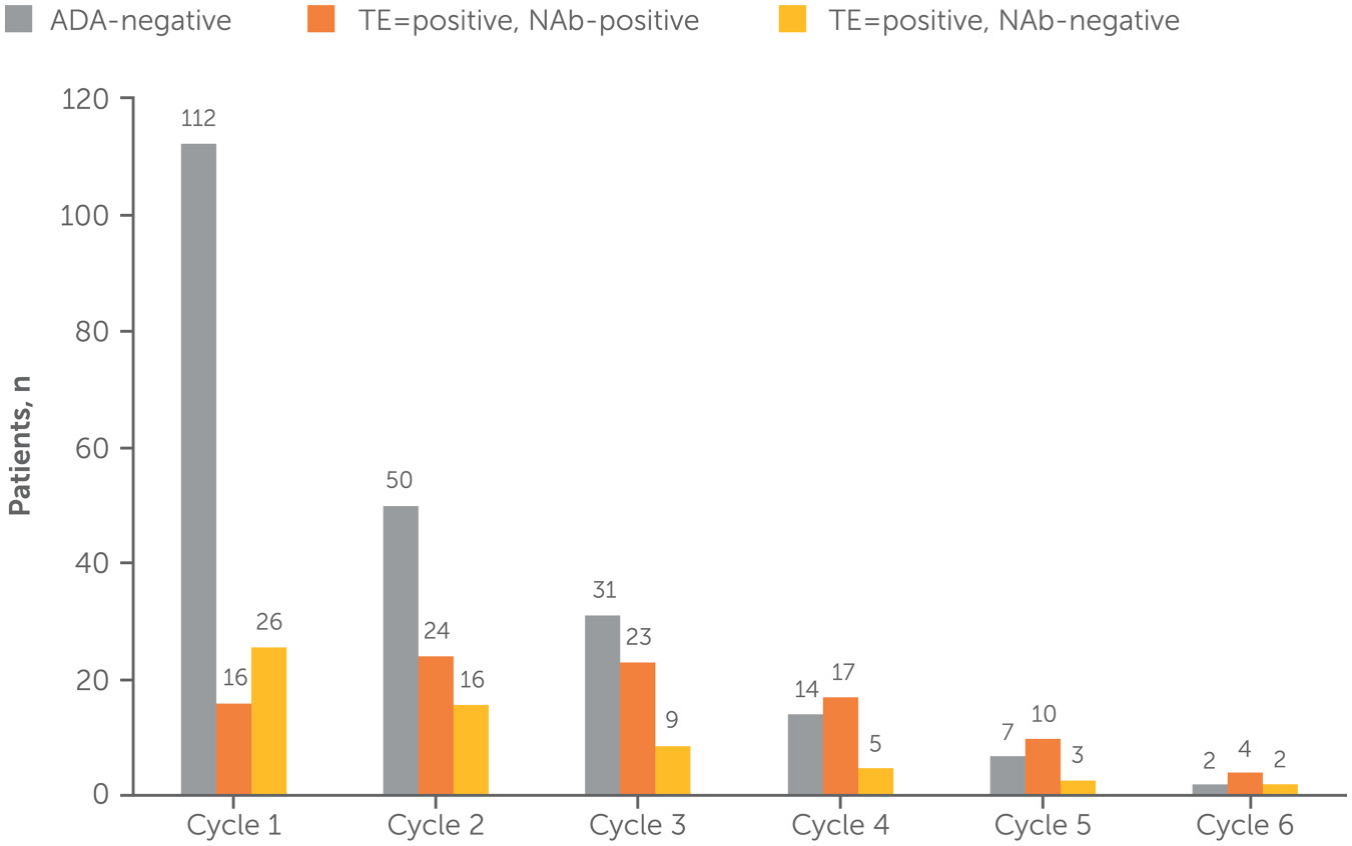
Overview of ADA category, by treatment cycle (immunogenicity pool).

A total of 168 patients received rozanolixizumab as a cyclic treatment regimen with no chronic weekly treatment interruption and were included in the immunogenicity pool; 155 (92.3%) patients were evaluable for ADAs (Figure 4). The incidence of TEAEs was generally consistent between ADA-positive (63/73, 86.3%) and ADA-negative (69/81, 85.2%) patients, with the most frequently reported TEAEs being headache (31 [42.5%] and 40 [49.4%] patients, respectively), diarrhea (18 [24.7%] and 19 [23.5%] patients) and pyrexia (14 [19.2%] and 11 [13.6%] patients). TEAEs that met the criteria for hypersensitivity reactions and injection-site reactions were also similar across both dose groups in the immunogenicity pool (data not shown).

The analysis period for each cycle is pre-treatment baseline to Day 43. ADA: anti-drug antibody; NAb: neutralizing antibody; TE: treatment-emergent.

## Discussion

This pooled analysis demonstrates that 6-week cyclic treatment with rozanolixizumab 7 mg/kg or rozanolixizumab 10 mg/kg was well tolerated in patients with gMG and had an acceptable safety profile that was consistent across repeated treatment cycles. The most common TEAEs across the two treatment groups were headache, diarrhea and pyrexia. The total incidence of all TEAEs was higher with rozanolixizumab 10 mg/kg than rozanolixizumab 7 mg/kg. Headache is a common adverse drug reaction associated with medications for treatment of gMG, including IVIg, and recurrence of headache is rare.^2,5,6^ However, the mechanism by which these headaches occur is unknown. Headache also occurred more frequently in female patients than in male patients, and in younger patients compared with elderly patients, which is consistent with reported sex and age differences in headaches in the general population.^9^ Unlike in clinical practice with other compounds, during gMG studies with rozanolixizumab, pre-medication for headache was not required. In case of a headache event the HCPs were advised to manage the event per standard of care.

One of 196 patients treated with rozanolixizumab in the gMG Phase 3 clinical program had drug-induced aseptic meningitis. There have been rare reports of aseptic meningitis in patients treated with IVIg and other monoclonal antibodies.^10–14^ The pathophysiology of rozanolixizumab-induced aseptic meningitis is not fully understood. The event resolved without sequelae and this is consistent with the clinical descriptions of aseptic meningitis.^15,16^

Patients with MG carry a higher risk of infection, including serious and severe infections, compared with the general population, which may be due to predisposing risk factors, including respiratory muscle weakness and prolonged immunosuppressive treatment.^17^ Approximately half of patients in this pooled analysis were receiving immunosuppressant treatment at baseline, which has been shown to lead to a greater risk of developing severe COVID-19 infection in patients with gMG.^18^ COVID-19 infection may increase the risk of developing an MG exacerbation, although this is not consistently observed in studies.^19–21^ As with all immunomodulatory therapies, there is a concern of increased risk of infection in patients receiving FcRn inhibitors due to its mechanism of action^22,23^; however, rozanolixizumab is not anticipated to impact quality of IgG or quantity of other Ig isotypes, or other cells of the innate and adaptive immune systems. The majority of infections observed during rozanolixizumab treatment in these studies were non-serious and mild-to-moderate in intensity, and the frequency of infections did not increase with repeated cyclic treatment; serious and severe infections were predominantly related to COVID-19.

While rozanolixizumab should not impact serum albumin concentrations due to distinct binding sites for IgG and albumin on FcRn,^4^ as a precaution, serum albumin levels were monitored during the studies. Minimal and not clinically meaningful reduction in mean albumin levels were noted, suggesting negligible impact of rozanolixizumab.

Repeated administration of monoclonal antibodies can elicit an immune response and formation of ADAs, which can interfere with a drug's ability to bind its target or alter pharmacokinetic and pharmacodynamic properties and lead to adverse immune reactions.^24^ As rozanolixizumab is a humanized IgG4 monoclonal antibody and does not contain or is conjugated to non-natural moieties or form multimeric complexes with FcRn,^4^ it is considered a low immunogenicity compound. Nonetheless, immunogenicity was monitored during rozanolixizumab treatment. There was no impact of immunogenicity on the safety of rozanolixizumab during this study.

As reported by Bril et al.,^8^ the results of the pooled efficacy analysis demonstrated consistent clinical improvements in measures of disease severity over repeated cycles of treatment. The pooled analysis reported here provides insight into the long-term safety of rozanolixizumab treatment. In addition, these safety data are in line with Phase 2 and 3 studies in chronic inflammatory demyelinating polyneuropathy and immune thrombocytopenia with no specific safety signals identified in gMG patients.^25–27^

## Conclusion

These pooled data, representing total rozanolixizumab exposure of 171.41 patient-years and including participants with more than 1 year of study participation and exposure of 121.1 patient-years, build on the positive results of the Phase 3 MycarinG study and demonstrate an acceptable safety profile of long-term rozanolixizumab treatment in patients with gMG that is consistent across repeated treatment cycles. This comprehensive review of available safety data confirms that rozanolixizumab is generally well tolerated and offers a convenient subcutaneous treatment option for patients living with AChR Ab+ or MuSK Ab+ gMG.
